# Functional genomic screening reveals asparagine dependence as a metabolic vulnerability in sarcoma

**DOI:** 10.1186/2194-7791-2-S1-A3

**Published:** 2015-07-01

**Authors:** Simone Hettmer, Anna C Schinzel, Daria Tchessalova, Nigel Richards, William C Hahn, Amy J Wagers

**Affiliations:** Howard Hughes Medical Institute, Department of Stem Cell and Regenerative Biology, Harvard Stem Cell Institute, and Joslin Diabetes Center, Harvard University, One Joslin Place, Boston, MA 02215 USA; Zentrum fuer Kinder- und Jugendmedizin, Universitaet Freiburg, Mathildenstrasse 1, 79106 Freiburg, Germany; Department of Medical Oncology, Dana-Farber Cancer Institute, Boston, MA 02115 USA; Department of Chemistry and Chemical Biology, Indiana University - Purdue University Indianapolis, IN USA

## Aims

Rhabdomyosarcomas (RMS) are mesodermal malignancies with skeletal muscle differentiation. The most common oncogenic mutations in RMS are in the RAS pathway. This study sought to identify actionable gene targets in sarcomas by selective targeting of the molecular networks that support the growth of *Ras*-driven sarcomas in mice.

## Methods

Mouse RMS tumors were induced by expression of oncogenic Kras(G12v) and disruption of CDKN2A (p16p19) using ex-vivo transduction and intramuscular injection of transduced mouse satellite cells. These sarcomas identified a cluster of genes upregulated in mouse sarcomas and human RMS compared to normal skeletal muscle. A customized shRNA proliferation screen was used to screen this gene cluster for actionable transcripts that reduced sarcoma cell proliferation. Target gene effects on sarcoma growth were evaluated in mouse and human RMS cell lines and xenografts.

## Results

Five immediately actionable proliferation-relevant gene targets were identified, and the anti-proliferative effects of 5 candidate chemicals, including asparaginase and amino sulfoximine (an inhibitor of asparagine synthetase, ASNS), were validated in mouse and human RMS cell lines. Silencing of ASNS, an amidotransferase that converts aspartate into asparagine, produced the strongest inhibitory effect on the growth of mouse *Kras; p16p19*^*null*^ sarcomas. ASNS silencing in mouse and human sarcoma cell lines reduced the percentage of S phase cells and impeded new polypeptide synthesis. These effects of ASNS silencing were reversed by exogenous supplementation with asparagine. Finally, genetic silencing of ASNS in mouse sarcoma cells combined with depletion of plasma asparagine inhibited tumor growth *in vivo*.

## Conclusions

The generation of new protein mass by rapidly proliferating sarcoma cells requires adequate Asparagine availability. Asparagine reliance of sarcoma cells may represent an actionable, metabolic vulnerability with potential anti-RMS therapeutic value.

